# Comparison of three different incision techniques in A1 pulley release on scar tissue formation and postoperative rehabilitation

**DOI:** 10.1007/s00402-016-2430-z

**Published:** 2016-02-29

**Authors:** Oliver Kloeters, Dietmar J. O. Ulrich, Gijs Bloemsma, Claire I. A. van Houdt

**Affiliations:** Department of Plastic Surgery, Radboud University Medical Center, Geert Grooteplein-Zuid 10, 6525 GC Nijmegen, The Netherlands; Department of Radiology, Radboud University Medical Center, Nijmegen, The Netherlands

**Keywords:** Trigger finger, A1 pulley release, Scar tissue, Skin incision, Surgical techniques, DASH, Outcome, Hand, Stenosing tenosynovitis

## Abstract

**Introduction:**

The optimal surgical approach for trigger finger release remains controversial in hindsight of postoperative rehabilitation as well as scar tissue formation. In this study, we comparatively evaluated the outcome of three different types of skin incision by employing the “Disability of the Arm Shoulder and Hand Score” (DASH) and by quantitative ultrasound measurements of scar tissue volume.

**Materials and methods:**

Thirty patients (32 triggerfingers) were enrolled in this study and randomly assigned to one of three groups: incision placed (1) transversal in distal palmar crease, (2) transversal and 2 mm distal from distal palmar crease, (3) longitudinally over MCP joint without crossing the distal palmar crease. Patients characteristics were noted and DASH scores were retrieved at four time points, (1) preoperatively (baseline), (2) 1 month, (3) 3 months, (4) 12 months postoperatively. Scar volume formation was assessed by ultrasound at 3 months postoperatively in 28 patients.

**Results:**

All groups showed a significant reduction in DASH values at 3 and 12 months postoperatively when compared to their own baseline levels. Group 3 showed the fastest and most pronounced reduction in DASH values at 1 month. Scar tissue formation was almost 57 % increased in group 1 vs group 2 and 3, however, not significant.

**Conclusion:**

There is no clear benefit of one incision technique over another. However, based on scar volume parameters, the significant faster recovery in the first month and the surgical ease of exposure and wound closure inclines us to favor the longitudinal incision (group 3) in future patients.

## Introduction

Stenosing tenosynovitis of a flexor tendon, also known as trigger finger, is a common debilitating hand pathology frequently seen and treated by hand surgeons [1, 2]. First described by Alphonse Notta in 1850, the name results from the painful popping or clicking while flexing/extending the involved digit [3]. This triggering is most often due to an inflammation-derived size discrepancy of the involved flexor tendons causing impingement at the level of the hypertrophic first annular (A1-) pulley [4]. If conservative treatment such as splinting and/or corticosteroid injection does not or no longer applies as a promising treatment option, surgical release of the A1-pulley is indicated [4, 5].

Especially in the field of handsurgery, the location and pattern of the incision with regard to hand function, anatomic considerations and aesthetics are paramount for the success of the surgery [6–8]. There is quite a plethora of different skin incisions described to approach the A1 pulley. The type of incision one surgeon will choose most likely depends on the surgical training and on his surgical mentors rather than having experienced and tested multiple incisions himself [9, 10]. Supposedly, this circumstance has led to strong convictions amongst some surgeons about which incision technique is superior over another.

Even though the A1-pulley release is considered as one of, and possibly the smallest, elective hand surgery procedure, most hand surgeons would agree that there is a considerable amount of patients that will present with a prolonged recovery period mostly due to scar formation along with subsequent irritation in daily activities or sports (e.g. golf, tennis). Adverse events between 5 and 36 % in the setting of trigger finger release have been reported [11–15], including persistent triggering, recurrence and wound healing problems such as infections, wound dehiscence, and painful scar tissue irritation. Anecdotic events involve tendon rupture, bowstringing, and nerve damage [16, 17]. However, by far the most reported complaints are wound healing irritations as well as pain and tenderness of overabundant scar tissue [11–13], limiting the patient’s use of his hand until weeks after the initial successful surgery.

In this prospective study, we hypothesized that the type of skin incision is a major predictor regarding the amount of postoperative scar tissue formation and the speed of recovery. Furthermore, there is no consensus recommendation on which incision technique is best for surgical release of trigger fingers. Therefore, we sought to investigate the level of scar tissue formation and degree of postoperative disability by comparing three of the most common incision techniques known to the authors: (1) horizontal at the level of the distal palmar crease, (2) horizontal 2–3 mm distal from the distal palmar crease, and (3) a longitudinal incision at the level of the A1-pulley location. We employed the “Disability of the Arm, Shoulder and Hand questionnaire” (DASH score) at different time points over a course of 12 months postoperatively and measured the volume of the resulting scar tissue quantitative ultrasound technique after 3 months [18].

## Materials and methods

### Inclusion of patients

Between January 2013 and February 2014, 30 patients (32 trigger fingers) were enrolled in this prospective and randomized observational study. A trigger finger was diagnosed by a board-certified hand surgeon based on patient history and physical examination (e.g. pain over the flexor tendon, tenderness or nodule over the A1 pulley, stiffness, and reproducible blocking or triggering with or without pressure at the A1-pulley while actively and passively flexing and extending the finger).

The inclusion criteria consisted of: age of 18 years or older, diagnosis of at least grade 2 trigger finger according to the Quinnell classification [19], duration of symptoms for at least 3 months, and absence of surgical treatment of the affected finger. Exclusion criteria involved triggering thumbs, more than one finger affected in one hand and a positive history of severe hand trauma.

Patients with diagnosed triggering of a finger who favored surgery over corticosteroid injection or who received unsuccessful corticosteroid injection(s) previously were scheduled for surgery. All patients provided a signed informed consent and approval by the local ethic committee was requested.

### Surgical technique

**Fig. 1:**
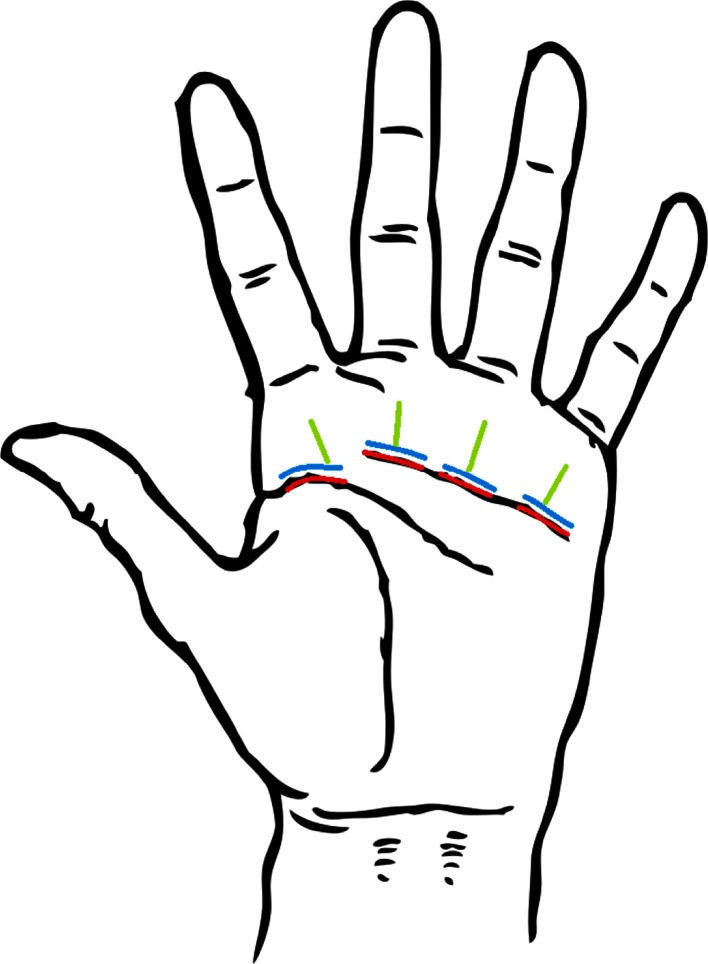
Schematic drawing of the three different incision techniques used in this study from D2 to D5

The included patients were randomly assigned, on the day of the surgery, to one of three operation techniques: (1) transversal in the distal palmar crease, (2) transversal 2–3 mm distally from distal palmar crease, and (3) longitudinally at the level of the A1-pulley without crossing the distal palmar crease proximal (Fig. 1). All surgeries were performed under local anesthesia, 3 cc of 1 % Xylocaine/Adrenalin (1:200,000). A tourniquet was placed at the forearm at 250 mmHg and a randomized incision pattern was carried out over a defined length of 15 mm. By longitudinal blunt dissection, the A1-pulley was identified and fully opened by a longitudinal incision over the pulley. Approximately 2–3 mm in width of the A1-pulley were resected to reduce the risk for recurrence. The skin was then closed with Prolene 4–0 and a circular bandage was applied to the hand and lower arm for 24 h postoperatively.

### Postoperative care

Directly after surgery, all patients were instructed to use the hand without any specific limitations. Two weeks postoperatively, the wound was evaluated again and the sutures removed.

### DASH scores

The ‘Disability of the Arm, Shoulder and Hand questionnaire’ (DASH) was used to measure functional outcome [20]. Patients were asked to fill in the DASH before surgery (baseline score) and at 1, 3 and 12 months after surgery. The DASH score was calculated for each time point and patient using the provided formula. Thirty patients were finally included in data analysis and 110 out of 128 possible DASH scores were received from all four time points resulting in a total response rate of 85.9 %.

### Ultrasound

**Fig. 2:**
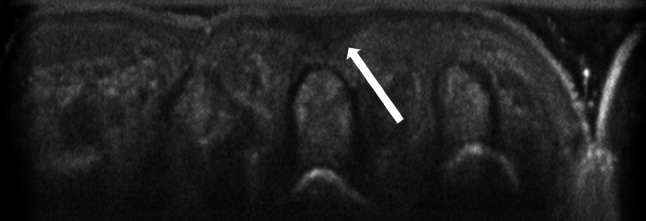
Representative picture of sonographic assessment for scar tissue. The *white arrow* indicates the triangular hypo-echoic scar tissue region

To quantify the amount of scar tissue we employed a ultrasound system (ACUSON S2000 ™, Siemens, Munich/Germany) 3 months (±1 week) after surgery to quantitatively measure the resulting scar volume. Scar tissue was clearly detectable and defined by a board certified radiologist as the subcutaneous hypo-echoic structure in the incision region throughout the complete length of the standardized incision length (Fig. 2). The hypo-echoic surface areas were measured and subsequently the scar volume calculated by a trained radiologist, blinded for the surgical techniques, using a Sectra workstation IDS7 version 15.1.32.3.

### Statistical analysis

All data are presented as mean ± SEM, with a *p* value of <0.05 considered to be significant. Statistical analysis was performed using SPSS (SPSS Statistics 20, IBM^®^, Amsterdam, The Netherlands). The differences between DASH scores of the three techniques were statistically compared with mixed models and multilevel linear regression analysis. The use of mixed models allowed for efficient comparison between longitudinal data, taking into account both the fixed effects of the experimental setup as well as the random effects of patients. For the comparison of the calculated volume ultrasound results, a univariate analysis of variance (one way-ANOVA) was employed to compare between the three surgical techniques.

## Results

Age and gender were evenly distributed in all three groups without significant differences with a mean age of 62 (±12.59) years. Thirteen women and 17 men were included. Twenty-four of 30 patients (80 %) were right-dominant and the affected fingers were evenly distributed on dominant and non-dominant hands [14 (44 %) vs 15 (47 %)], (Table 1). Furthermore, about one out of three affected fingers previously received corticosteroid injection, six fingers once, two fingers twice and one finger three times (Table 1).

**Table 1:** Demographic characteristics of included patients

	*N*	%^a^
Number of patients	30	
Age (mean ± SD in years)	61.77 ± 12.59	
Gender (male/female)	13/17	43/57
Dominance (right/left/unknown)	24/3/3	80/10/10
Comorbidities		
Carpal tunnel syndrome	4	11
Cardiovascular	4	11
Pulmonary	3	11
Diabetes mellitus type 2	1	7
Other^b^	7	25
None	7	14

	*N*	%^c^
Number of operated digits	32	
Operated on dominant hand		
Yes	14	44
No	15	47
Unknown	3	9
Previous corticosteroid injection		
No	19	59
Yes	10	31
Once	6	
Twice	2	
Three times	1	
Yes but unknown amount	1 3	9
Unknown		

^a^Percentage is calculated by number of patient with the comorbidity divided by 30 patients

^b^Other comorbidities include fibromyalgia, radicular pain syndrome, extravasation of chemotherapeutic drugs in lower arm, glaucoma, hemophilia, thrombocytopenia, kidney stones

^c^Percentage is calculated by number divided by 32 digits

**Fig. 3:**
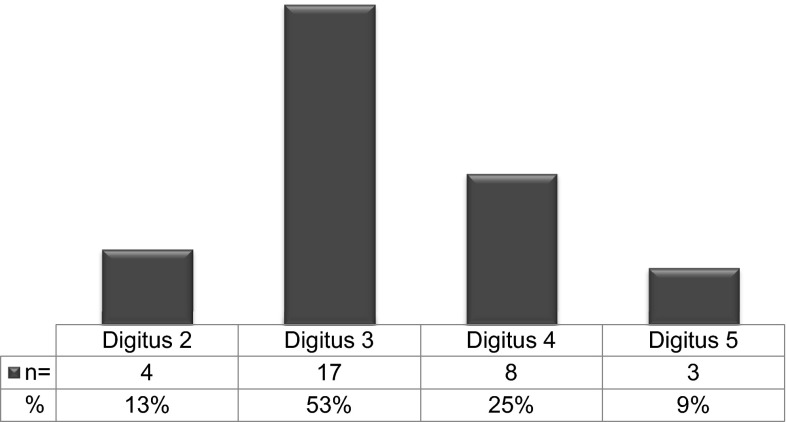
Distribution of operated digits (*n* = 32 digits)

The third finger was most often affected (*n* = 17; 53 %), followed by the ring finger (*n* = 8; 25 %), then index finger (*n* = 4; 13 %) and finally small finger (*n* = 3; 9 %), (Fig. 3).

We have received and included 110 complete DASH score sheets from all four time points of 128 possible DASH scores, resulting in an overall response rate of 85.9 % (Table 2).

**Table 2:** Percentages of retrieved DASH scores per time point and group

Group	1	2	3	All
*N*	11	10	11	32
Baseline	90.9 %	100 %	100 %	96.9 %
1 month	81.8 %	90.0 %	100 %	90.6 %
3 months	100 %	70.0 %	81.8 %	84.4 %
12 months	72.7 %	63.6 %	72.7 %	71.9 %
Total	86.4 %	80.9 %	88.6 %	85.9 %

*Group 1* transverse in distal palmar crease, *2* transverse 2 mm distal from distal palmar crease, *3* longitudinal

### DASH scores

**Fig. 4:**
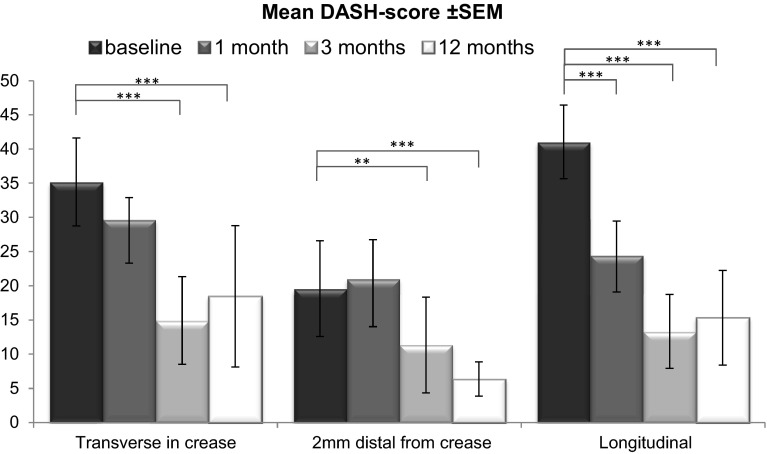
DASH scores at different time points in mean ± SEM per group, comparing the baseline to postoperative time points; **significant difference of *p* < 0.01 and ***p < 0.001

**Fig. 5:**
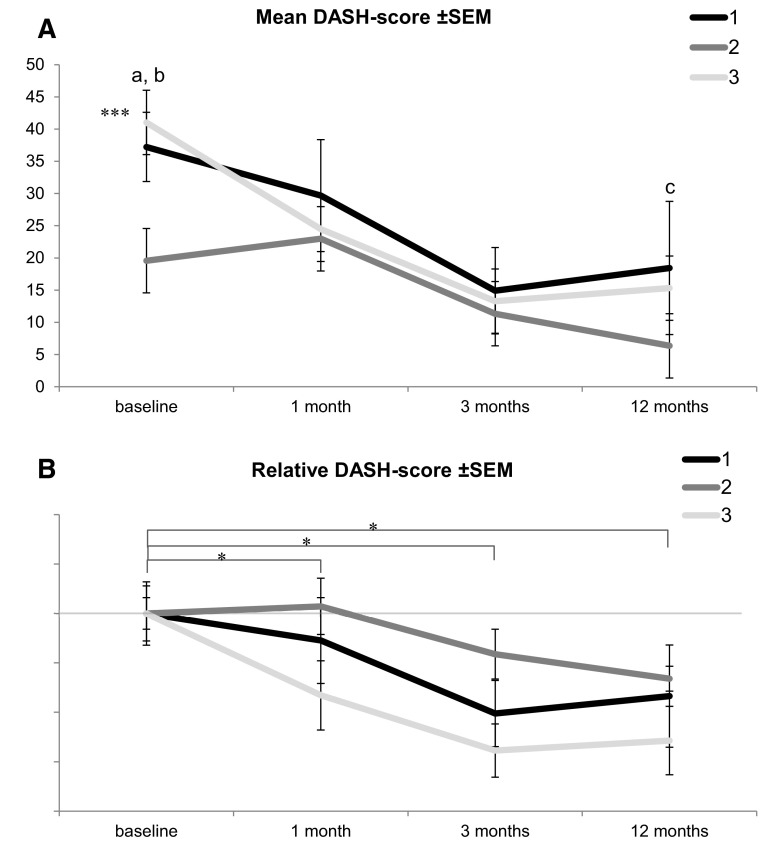
**a** Mean DASH scores at different time points ± SEM per group; *a* significant difference between group 1 and 2, *p* < 0.001; *b* significant difference between group 2 and 3, *p* < 0.001; *c* significant difference between group 1 and 2, *p* < 0.05. **b** Relative differences (delta) of mean DASH scores over time, each delta comparing between two groups; *significant difference, *p* < 0.05, between groups 2 and 3 with a significant faster decrease for group 3; *group 1* transverse in distal palmar crease, *2* transverse 2 mm distal from distal palmar crease, *3* longitudinal; ***p < 0.001

We measured DASH scores at preoperative baseline as wells as at 1, 3, and 12 months postoperatively (Table 3; Fig. 4). Noteworthy, there was a significant difference in baseline DASH scores between group 2 vs group 1 and 3 (Fig. 5a).

**Table 3:** Mean ± SEM per group for each time point

Group	Baseline	1 month	3 months	12 months
1	**37.2** ± 5.38	**29.7** ± 8.69	**14.9** ± 6.71	**18.4** ± 10.33
2	**19.6** ± 3.19***	**23.0** ± 5.70	**11.3** ± 5.03	**6.4** ± 2.51
3	**41.0** ± 6.41	**24.5** ± 7.00	**13.3** ± 5.39	**15.3** ± 6.93

*Group 1* transverse in distal palmar crease, *2* transverse 2 mm distal from distal palmar crease, *3* longitudinal

*** Significant difference between group 2 vs 1 and 3 with *p* < 0.001

We were able to measure highly significant differences in DASH score values in all groups when baseline DASH scores were compared to all different time points postoperatively: DASH scores in group 1 dropped from baseline (DASH score: 37.2) by 7.5 points 1 month postoperatively, to postoperative month 3 by 22.3 points (*p* < 0.001) and reached after 1 year 18.8 points (*p* < 0.001). Group 2 had a significant lower baseline level (DASH score: 19.6) then group 1 and 3 and increased at postoperative month 1 by 3.4, declined by postoperative month 3 by 8.3 (*p* < 0.01) and was at 12 months at 13.2 points (*p* < 0.001). Finally, group 3 declined from baseline (DASH score: 41.0) to 1 month postop by 16.5 (*p* < 0.001), to postop month 3 by 27.7 (*p* < 0.001) and after 1 year by 25.7 points (*p* < 0.001) (Fig. 4).

DASH scores were also compared in between groups at different time points. We found a significant difference in baseline values in group 2 vs group 1 and 3 (*p* < 0.001). Finally, 1 year after surgery, patients in group 2 had a significant lower DASH score than patients in group 1 (*p* < 0.05) (Fig. 5a).

In addition, we investigated for relative DASH score changes (delta-DASH) in all time segments versus baseline values and compared them to the corresponding remaining two groups to determine the speed of recovery. We found a significant (*p* < 0.05) difference in all time segments when compared to baseline values between group 2 and 3 (Table 4; Fig. 5b).

**Table 4:** Relative differences (delta) of mean DASH scores over time, each delta comparing between two groups

Group	Months from 0 to 1	0 to 3	0 to 12	1 to 3	1 to 12	3 to 12
1 to 2	10.4	13.6	12.8	3.2	2.4	−0.8
1 to 3	−9.3	−7.4	−4.2	1.8	5.1	3.3
2 to 3	−19.7*	−21.1*	−**17.0** ^*^	−1.4	2.7	4.1

*Group 1* transverse in distal palmar crease, *2* transverse 2 mm distal from distal palmar crease, *3* longitudinal

* Significant difference between groups 2 and 3 with *p* < 0.05

### Scar volume

**Fig. 6:**
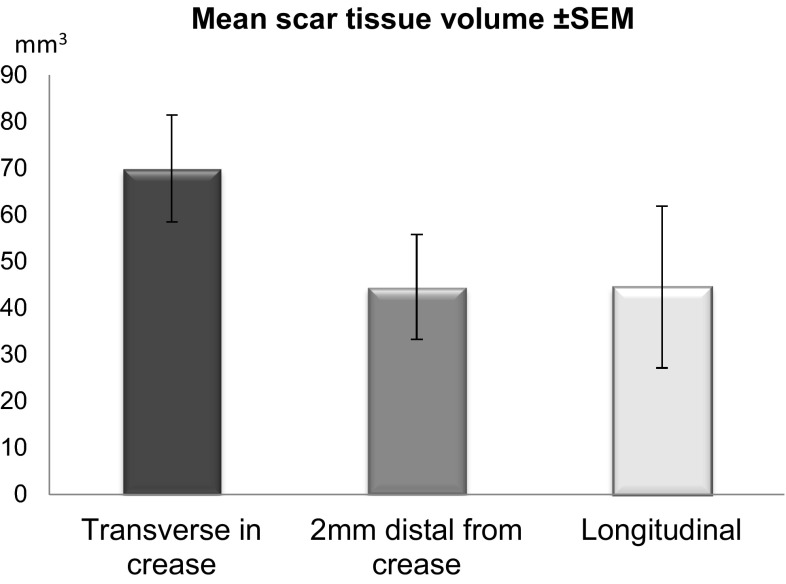
Volumes of scar tissue in mm^3^ (mean +SEM) per group; there were no significant differences between groups

We measured the scar volume (in mm^3^) using high-resolution ultrasound 12 weeks postoperatively in 28 incisions. Although scar volume was markedly higher in group 1 (69.98 ± 11.49) we could not detect a significant difference when compared to group 2 (44.53 ± 11.23) and group 3 (44.55 ± 17.41), (Fig. 6).

## Discussion

Here, we investigated the effect of three different skin incisions to access the A1-pulley and their impact on postoperative outcomes including the DASH score as well as quantitative scar volume measurement. The total response rate of DASH questionnaires was 85.9 % allowing for reliable data analysis. In this study, the middle finger was most often affected (61 %), followed by the ring finger (29 %) and index finger (14 %). This pattern of distribution is in line with findings from other investigators [4].

About 30 % of our patients received at least once a corticosteroid injection as a semi-invasive treatment for A1-pulley stenosis. Although the aim of this study was not to investigate the effectiveness of corticosteroids, one can well conclude that a relevant amount of patients requires surgery after initial unsuccessful injection(s), which has been demonstrated extensively by other authors [21–27].

Even though scar volume was elevated by almost 75 % in group 1 3 months post surgery compared to groups 2 and 3, these changes were statistically not significant. However, this increase in group 1 should not be neglected in terms of interpretation. We are inclined to hypothesize that an incision placed directly into a fibroseptal-anchoring structure such as the distal palmar crease stimulates a more pronounced scar tissue reaction compared to incision techniques outside this crease. This might be caused by the local disruption and the subsequent cellular stimulation of this mechanically relevant structure with its predominance of connective tissue. Furthermore, an everted wound closure technique was more complex in group 1 due to the inverted nature of the distal palmar crease. Groups 2 and 3 showed almost identical scar tissue volume levels which can be interpreted as another indicator that an incision located in the crease appears to be the most denominating factor for subsequent scar tissue formation.

All types of incisions and A1 pulley releases caused a significant improvement of symptoms when looking at DASH scores at 3 and 12 months. The most pronounced and fastest amelioration of symptoms were found in group 3 (longitudinal incision) indicated by a highly significant reduction of the DASH score already at 1 month postoperatively (*p* < 0.001). At this early time point, group 2 even exerted a slight increase and group 1 only a mild reduction (Figs. 4, 5a, b). We tend to explain these findings by the nature of the longitudinal incision: (1) excellent exposure of the A1 pulley, (2) facilitated everted wound closure and (3) tension forces parallel to the incision as opposed to scar stimulating perpendicular tension forces in horizontal incisions (group 1 and 2) when the fingers are extended. A slight disadvantage of the longitudinal incision might be its limitation in distal and proximal direction for the rare necessity to lengthen the incision. These circumstances taken together lead in our opinion to lesser tissue irritation and therefore accelerated wound healing with less scar formation in an early stage of wound healing.

To our surprise, we found a significant difference in the baseline DASH scores: group 1 and 3 demonstrated a significant higher baseline DASH score compared to group 2. This is clearly a coincidental flaw in this study and could have been prevented if patients would have been matched for DASH values instead of age and gender. However, this might have led to significant differences in age and/or gender distribution and additionally would have limited the blinded and randomized nature of this study. This being said, it stands to reason if the significant reduction in DASH score values of group 2 vs group 1, 12 months postoperatively, is of any clinical value.

Finally, we analyzed relative DASH score changes (delta-DASH) at all time points in all groups. The degree of reduction in DASH score level was significantly increased at all postoperative time points when group 3 was compared to group 2; however, not significant when group 3 was compared to group 1 (Fig. 5b, *p* < 0.05). These findings would also confirm our idea of a supremacy of the longitudinal incision over the incision 2 mm distal from the distal crease.

In summary, our findings in this study suggest an incision at the level of the distal crease as least favorable given the increased levels of scar tissue formation and the obvious surgical difficulties for adequate wound closure when compared with the two other incisions. However, after 3 and 12 months DASH scores dropped as significantly as in group 3. When finally comparing group 2 vs 3 with the aim to determine the best incision we are inclined to favor the longitudinal incision given the outstanding early first month results with a highly significant drop in DASH score values.
